# Cumulative experiences of racism and mental health outcomes in Colorado minority adolescents

**DOI:** 10.3389/fpubh.2026.1781495

**Published:** 2026-05-28

**Authors:** Sumaita Choudhury, Ashley Brooks-Russell, Erin Wright-Kelly

**Affiliations:** Injury and Violence Prevention Center, Colorado School of Public Health, University of Colorado Anschutz Medical Campus, Aurora, CO, United States

**Keywords:** adolescents, experiences of racism, mental health, racial and ethnic minorities, suicide

## Abstract

**Background:**

Suicide is a leading cause of death for adolescents. Research has demonstrated that experiencing racism can disrupt adolescent development and negatively impact mental health, particularly among racial and ethnic minorities.

**Aims:**

We examined how the cumulative impact of multiple experiences of racism was associated with mental health outcomes including depressive symptoms and suicide ideation among high school adolescents who identified within a racial or ethnic minority group.

**Methods:**

This cross-sectional study used the 2023 Healthy Kids Colorado Survey data (*N* = 49,989). We estimated the weighted prevalence of the five individual racism experiences and four mental health outcomes by race and ethnicity. Finally, we conducted a series of weighted adjusted multivariable logistic regression models stratified by three racial and ethnic minority groups (Asian American, Black/African American, and Hispanic/Latinx), to assess the associations between the cumulative experiences of racism and the mental health outcomes.

**Results:**

Across the three racial and ethnic groups, Asian American adolescents reported the highest likelihood of considering suicide and attempting suicide (aOR: 7.8; 95% CI: 2.7, 22.5), Black/African American adolescents reported significantly more robust association with depressive symptoms (aOR: 4.1, 95% CI: 2.1, 8.1), and Hispanic/Latinx adolescents reported significantly more robust association with making a suicide plan (aOR: 4.9, 95% CI: 3.3, 7.4).

**Conclusion:**

Our findings showed that among Asian, Black, and Hispanic Colorado high schoolers with three or more experiences of racism had significantly higher odds of reporting poor mental health, highlighting the need for school and community efforts to implement anti-racism policies and interventions.

## 1 Introduction

Racism is widely recognized as a chronic psychosocial stressor, and repeated experiences of discrimination may accumulate over time, contributing to sustained psychological distress and adverse mental health outcomes (1, 2). Even everyday experiences of unfair treatment, through their frequency and persistence, may produce levels of distress comparable to more overt discriminatory events. Racism negatively impacts the health and quality of life of racial and ethnic minority groups throughout adolescence and adulthood (3, 4). Repeated experiences of racism are a result of systemic inequalities, societal stigma, discrimination, marginalization, and unequal access to resources and opportunities based on race/ethnicity (3, 5). Adolescence is a crucial time for developing critical cognitive skills, self-esteem, autonomy, and neurodevelopment that help shape the foundation for later stages of life (6). Research has demonstrated that experiencing racism can hinder the developmental process, which has been shown to negatively affect adolescents’ mental health and can lead to depression, psychological distress, suicide ideation, suicide plans, and suicide attempts among these populations (6–9). Recognizing racism as a cumulative stressor may help clinicians and policymakers better understand how repeated exposures contribute to mental health disparities among adolescents.

Suicide is a leading cause of death for adolescents ages 10–19 years (10). There are substantial disparities in suicide rates among adolescents across race and ethnic groups. In 2002, among adolescents (10–19), those who identified as American Indian and Alaska Native died by suicide at a rate of 12.1 per 100,000, nearly twice that of White adolescents (6.3 per 100,000), and Black adolescents (6.0 per 100,000;) (10). Suicide rates were lower for adolescents who identified as Asian American (4.7 per 100,000), Hispanic (independent of race; 4.4 per 100,000), and multiple racial or ethnic identities (4.1 per 100,000) (10). From 2018 to 2022, Black adolescents (10–19) were the only racial subgroup to experience an increased suicide rate, from 5.1 to 6.0 per 100,000 (19% increase) (10).

Adolescents and young adults who have experienced racism are more likely to engage in risky behaviors including alcohol and marijuana use, risky sexual behaviors, delinquency, and aggression (9). Studies have found that the cumulative impact of multiple racism experiences is significantly associated with depression and suicidality (11, 12). Additionally, studies have also reported that Black adolescents experienced the highest levels of racism, followed by Asian Americans (6, 7). However, most of the existing studies regarding the link between racism and suicidality examined Black youth and emerging adults with limited focus on other racial and ethnic minority groups and high-school aged adolescents (12–15). Finally, many studies assessing the relationship between racism and mental health have small sample sizes limiting generalizability to the general population (11–15).

Schools across Colorado provide a range of school-based mental health supports, including school counselors, psychologists, and partnerships with community behavioral health providers, guided by the state’s framework for comprehensive school behavioral health services (16). Additionally, Colorado supports school mental health through initiatives such as Project Advancing Wellness and Resiliency in Education (AWARE), School Mental Health Support programs (SMHSP), and partnerships with community providers to address issues like depression, anxiety, and suicidality among students. Schools also operate under statewide policies addressing bullying and discrimination to promote safe and supportive learning environments [Student Learning Division/Teaching and Learning Unit/Office of Learning Supports (17)]. While there are numerous programs and strategies in place to support mental health of Colorado adolescents; little is known about how well these efforts have supported the mental health of racial and ethnic minority students and if there are any gaps that would better support these sub-populations of Colorado adolescents. Disparities in mental health outcomes related to experiencing racism still persist, highlighting the importance of examining how experiences of racism may contribute to these outcomes and identifying potential areas for school mental health policy improvements. Taken together, additional research using representative data is needed to understand the impact of experiencing racism on mental health outcomes among high school-aged adolescents across diverse racial and ethnic minority groups.

The purpose of this study was to examine the prevalence of experiencing racism among high school-aged adolescents who identified within a racial or ethnic minority group, and associations with adverse mental health outcomes including depression and suicide and analyze how the cumulative impact of multiple self-reported racism experiences was associated with mental health outcomes. Moreover, this study expanded on previous research that relied on a single measure of experiencing racism (15, 18) and included multiple items measuring experiences of racism.

## 2 Methods

### 2.1 Study design and data collection

Data were from the 2023 Healthy Kids Colorado Survey, an anonymous biennial survey, administered online in a sample of public middle and high schools in Colorado. The present analysis includes only high school data. This decision aligns with the study’s purpose of examining experiences of racism and associated mental health outcomes specifically among high school-aged adolescents from racial or ethnic minority groups. High school students were selected because suicide is reported as the third leading cause of death in this population (19). Furthermore, the sampling approach to the middle and high school samples were different, with the high school sample utilizing a regional stratified sample and the middle school sample, which is less than a quarter of the size of high school, using a statewide-only sample. These differences make it complicated to combine them and limits the utility of the smaller middle school sample when examining differences in subgroups. Schools were sampled using a 2-stage stratified cluster design by using a sampling frame of all public high schools in Colorado. The survey was administered online, in classrooms during a regular class period. Participation was voluntary and approved by parents. The study was approved by the Colorado Multiple Institutional Board.

### 2.2 Measures

The HKCS instrument consists primarily of questions from the Centers for Disease Control and Prevention’s (CDC) Youth Risk Behavior Surveillance System (YRBS) (20), with additional questions added from validated sources including optional questions recommended for state administrations of the YRBS.

### 2.3 Outcome variables

#### 2.3.1 Depressive symptoms

Depression is a mental health disorder causing feelings of hopelessness, sadness, functional impairment and loss of interest (21). Depressive symptoms was assessed by a single item “*during the past 12 months, did you ever feel so sad or hopeless almost every day for 2 weeks or more in a row that you stopped doing some usual activities?*” with binary response options “yes” or “no.”

#### 2.3.2 Considered suicide

Consider attempting suicide refers to an individual considering or thinking about suicide (22). It was measured by a single item “*during the past 12 months, did you ever seriously consider attempting suicide?”* with binary response options “yes” or “no.”

#### 2.3.3 Suicide plan

Suicide plan refers to the development of a specific and organized approach for attempting suicide, often involving details such as the method, timing, location, and access to means (23). It was assessed by a single item “*during the past 12 months, did you make a plan about how you would attempt suicide?*” with dichotomized response options “yes” or “no.”

#### 2.3.4 Suicide attempt

Suicide attempt refers to an intentional, self-inflicted act undertaken with the aim of ending one’s life, regardless of whether it leads to harm or death (22). It was measured using a single item “*during the past 12 months, how many times did you actually attempt suicide?*” with response options ranging from “0 times” to “6 or more times.” This study collapsed the responses into binary response options “0 times” or “1 or more times.” Responses were collapsed into a binary variable because higher-frequency categories had small cell sizes, and dichotomization improved statistical stability in the regression analyses.

To facilitate readability, we renamed each of the mental health variable as follows, “depressive symptoms,” “considering suicide,” “making a suicide plan,” and “attempting suicide.”

### 2.4 Independent variables

#### 2.4.1 Experiences of racism

The experiences of racism questions were from the CDC YRBS’s 2021 optional questions (24). Racism was originally evaluated by a “check all that apply” item, which included five racism-related categories. The responses as follows, and the variable-label: ‘racism none’ (*“I did not experience any racism”*), ‘treated unfair’ (*“Treated unfairly or badly in school because of your race or ethnicity”*), ‘followed by security’ (*“Watched closely or followed around by security guards or store clerks at a store or mall because of your race or ethnicity”*), ‘assumed less intelligent’ (*“People assumed you are less intelligent because of your race or ethnicity”*), and ‘family treated unfairly’ (*“Seen your parents or other family members treated badly or unfairly because of the color of their skin, language, accent, or because they are from a different country or culture”*). To facilitate readability, we renamed each of the racism variable as follows, “no racism experience,” “treated unfairly,” “followed by security,” “presumed less intelligent,” and “family treated unfairly.”

To estimate the prevalence of each racism experience in our sample, we created separate non-mutually exclusive dichotomized racism variables for the five categories mentioned above with response options “yes” or “no.” For our adjusted multivariable regression models, we created a cumulative score for each participant by adding the total number of racism experiences from the four racism categories (i.e., treated unfairly, followed by security, presumed less intelligent, and family treated unfairly) with scores ranging from 0 to 4. Participants with a score of 0 were categorized as “no racism experience,” a score of 1 was categorized as “one experience of racism,” a score of 2 was categorized as “two experiences of racism,” and a score of 3 or higher was categorized as “three or more experiences of racism.” Due to small sample size among participants reporting either three or four experiences of racism, these responses were combined into a single “three or more experiences” category to ensure adequate statistical power and regression estimates.

#### 2.4.2 Race

Race was initially assessed using a “check all that apply” item with multiple categories. We subsequently created a race variable with mutually exclusive categories, including American Indian/Alaska Native, Black/African American, Asian American (includes Southeast Asian and Asian), Hispanic/Latinx, Middle Eastern/North African/Arab, Native Hawaiian/Other Pacific Islander, White, another identity, and Multiracial (includes individuals who selected two or more categories). As the original survey allowed respondents to select multiple racial identities, mutually exclusive racial categories were created for analysis. Adolescents selecting more than one racial category were classified as multiracial; however, due to the heterogeneity of racial identity combinations and limited sample sizes for specific multiracial subgroups, analyses focused on the primary racial and ethnic groups with sufficient sample sizes for stable estimates. Analytic decisions regarding how to classify multiracial respondents are common in population-based research, as incorporating multiracial identities into traditional racial frameworks presents methodological challenges (25).

#### 2.4.3 Covariates

Demographic factors that were adjusted for in this study include gender (male, female, or other (non-binary, another identity, and not sure)), grade (collapsed 9th-10th grade and11th–12th grade), and sexual orientation (heterosexual, sexual minorities (gay or lesbian, pansexual, or asexual), or other (another identity and not sure)).

#### 2.4.4 Statistical analyses

We conducted all statistical analyses using R Studio software version 2024.04.2 and STATA/BE version 17.0 (18, 26). First, we examined the unweighted descriptive statistics of our study sample and reported the frequencies and percentages for the demographic factors. Second, we estimated the weighted prevalence of the five individual racism experiences (i.e., no racism experience, treated unfairly, followed by security, presumed less intelligent, and family treated unfairly) and the weighted prevalence of four mental health outcomes (depressive symptoms, considered attempting suicide, suicide plan, suicide attempt) by race and reported the weighted prevalence and 95% confidence intervals (CI), and tested for differences using the Rao-Scott *χ*^2^ tests (27).

Finally, we conducted a series of weighted adjusted multivariable logistic regression models stratified by three racially/ethnic minority groups (limited to the groups with sufficiently large sample sizes to ensure robust analysis), including Asian American, Black/African American, and Hispanic/Latinx to assess the associations between the cumulative experiences of racism and the mental health outcomes. We reported adjusted odds ratios (aOR) and 95% CI for all the regression models and the significance level was set at *p* < 0.05.

## 3 Results

### 3.1 Demographic characteristics

The demographic characteristics, including unweighted frequencies and percentages are presented in Supplementary Table 1. Overall, 49,989 high school-aged adolescents participated in the survey. Most were male (48.6%), 9th-10th graders (56.6%), White (50.3%), and heterosexual (75.9%).

### 3.2 Prevalence of each racism experience across demographic factors

Table 1 displays the weighted prevalence of each racism experience among our sample. Among racial and ethnic groups, Native Hawaiian/Other Pacific Islander adolescents had the highest rates of racism related to being treated unfairly, assumed less intelligence, and family treated unfairly. However, this group had a limited sample size to permit further analyses. Black/African Americans reported the highest prevalence of racism experiences for being presumed less intelligent (21.3; 95% CI: 17.3, 26.0), followed by security (20.1%; 95% CI: 16.9, 23.8), and being treated unfairly (12.7, 95% CI: 9.4, 17.0). Asian American adolescents reported higher prevalence of their family being treated unfairly (23.4%; 95% CI:19.3, 28.1).

**Table 1:** Weighted prevalence of self-reported experiences of racism by race and ethnicity.

Experiences of racism					
Race variables	No racism experience	Treated unfairly	Followed by security	Presumed less intelligent	Family treated unfairly
	% [95% CI]	% [95% CI]	% [95% CI]	% [95% CI]	% [95% CI]
Overall	84.0%	5.0%	6.2%	6.6%	9.1%
Race					
American Indian/Alaska Native	86.6 [76.9, 92.7]	4.5 [2.03, 9.7]	6.3 [2.7, 13.9]	5.1 [2.4, 10.4]	3.3 [1.6, 6.8]
Black/African American	60.2 [55.4, 64.8]	12.7 [9.4, 17.0]	20.1 [16.9, 23.8]	21.3 [17.3, 26.0]	19.9 [16.3, 24.0]
Asian American	67.6 [62.6, 72.2]	11.2 [9.1, 13.7]	7.9 [4.8, 12.7]	5.7 [4.4, 7.3]	23.4 [19.3, 28.1]
Hispanic/Latinx	74.6 [72.1, 77.0]	6.1 [4.9, 7.6]	10.0 [8.7, 11.4]	10.4 [9.0, 12.0]	15.5 [14.1, 17.1]
Middle Easter/North African/Arab	59.4 [53.1, 65.3]	10.8 [7.1, 16.0]	10.9 [7.6, 15.8]	8.3 [5.3, 13.2]	31.0 [25.6, 37.1]
Native Hawaiian/Other Pacific Islander	50.1 [33.2, 67.1]	21.7 [8.8, 44.5]	13.6 [3.1, 44.2]	31.5 [11.0, 63.1]	32.4 [23.9, 42.2]
White	96.1 [95.5, 96.6]	2.04 [1.7, 2.5]	0.8 [0.6, 1.0]	1.5 [1.2, 1.7]	1.3 [1.1, 1.5]
Another Identity	69.6 [57.1, 79.7]	12.5 [7.5, 20.0]	13.7 [7.3, 24.2]	15.1 [9.0, 24.0]	12.9 [5.4, 27.9]
Multiracial	73.2 [71.4, 74.9]	9.4 [8.17, 10.8]	13.1 [11.7, 14.7]	11.4 [9.77, 13.2]	15.7 [14.6, 16.8]

The table includes all weighted row percentages and 95% CI for all the variables and weighted frequencies across the racism variable. For the gender variable, the option “Other” includes (non-binary, another identity, and not sure). For the sexual orientation variable, the option “Other: includes (another identity and not sure). Race and ethnicity is a mutually exclusive variable. Sample sizes vary due to missing data ~ < 5%.

### 3.3 Prevalence of suicide related outcomes across demographic factors

Table 2 displays the weighted prevalence of mental health outcomes among our sample. Across racial and ethnic groups, the highest rates of mental health outcomes were observed in those with smaller sample sizes and wider confidence intervals (American Indian/Alaskan Native, Native Hawaiian/Other Pacific Islander, and Middle Eastern/North African/Arab). Asian adolescents’ reporter higher prevalence of considering suicide (10.4; 95% CI: 7.2, 15.0), making a suicide plan (10.2; 95% CI: 7.5, 13.8), and attempting suicide (5.8; 95% CI: 4.1, 8.0). Conversely, Hispanic/Latinx adolescents reported higher prevalence of depressive symptoms (27.1, 95% CI: 25.8, 28.3).

**Table 2:** Weighted prevalence of mental health outcomes by race.

Mental health outcomes				
Race variables	Depressive symptoms	Considering suicide	Making a suicide plan	Attempting suicide
	% [95% CI]	% [95% CI]	% [95% CI]	% [95% CI]
Overall	26.0%	11.1%	9.2%	5.4%
Race				
American Indian/Alaska Native	41.8 [26.2, 59.2]	17.5 [8.7, 32.0]	15.0 [6.8, 30.1]	11.7 [6.7, 19.8]
Black/African American	25.0 [21.9, 28.4]	9.4 [7.8, 11.3]	8.0 [6.6, 9.7]	4.8 [3.7, 6.2]
Asian American	26.2 [21.0, 32.2]	10.4 [7.2, 15.0]	10.2 [7.5, 13.8]	5.8 [4.1, 8.0]
Hispanic/Latinx	27.1 [25.8, 28.3]	8.7 [8.0, 9.5]	7.1 [6.5, 7.9]	5.4 [4.6, 6.3]
Middle Easter/North African/Arab	26.5 [21.1, 32.8]	8.8 [4.5, 16.7]	10.7 [6.4, 17.4]	9.07 [6.0, 13.5]
Native Hawaiian/Other Pacific Islander	26.4 [15.3, 41.5]	14.1 [7.2, 25.7]	6.3 [2.9, 13.2]	9.4 [4.2, 19.9]
White	23.5 [22.4, 24.6]	12.4 [11.2, 13.6]	10.2 [9.3, 11.2]	5.0 [4.3, 5.8]
Another Identity	29.9 [22.1, 39.1]	14.0 [9.0, 21.3]	13.1 [8.0, 20.8]	6.3 [3.8, 10.1]
Multiracial	31.8 [29.5, 34.2]	15.4 [12.9, 18.3]	12.3 [11.1, 13.5]	7.5 [5.9, 9.4]

The table includes all weighted row percentages and 95% CI for all the variables and weighted frequencies across the racism variable. For the gender variable, the option “Other” includes (non-binary, another identity, and not sure). For the sexual orientation variable, the option “Other: includes (another identity and not sure). Race is a mutually exclusive variable. Sample sizes vary due to missing data ~ < 5%.

### 3.4 Adjusted multivariable regression models

In this section, we present the adjusted regression findings related to the experiences of racism and mental health outcomes among racial and ethnic minority groups.

### 3.5 Asian American

The adjusted multivariable results demonstrated significant associations between one, two, and three or more experiences of racism across most of the four mental health outcomes among Asian American adolescents (Table 3). Adolescents with three or more experiences of racism exhibited stronger significant associations with depressive symptoms (aOR: 3.9; 95% CI: 1.8, 8.6), considering suicide (aOR: 7.8; 95% CI: 2.7, 22.5), making a suicide plan (aOR: 4.9; 95% CI: 2.3, 10.6), and attempting suicide (aOR: 6.7; 95% CI: 2.3, 19.5). Furthermore, across the three racial and ethnic groups, Asian American adolescents reported the highest likelihood of considering and attempting suicide.

**Table 3:** Adjusted multivariable logistic regression regarding the association between cumulative experiences of racism and mental health outcomes among Asian American adolescents.

Asian American mental health outcomesw								
Experience of racism variables	Depressive Symptoms		Considering Suicide		Making a Suicide Plan		Attempting Suicide	
	aOR^a^	95% CI	aOR^a^	95% CI	aOR^a^	95% CI	aOR^a^	95% CI
Experience of racism								
No racism experience	Ref.	Ref.	Ref.	Ref.	Ref.	Ref.	Ref.	Ref.
One racism experience	3.7***	[2.0, 7.2]	3.6***	[1.8, 7.2]	2.8***	[1.8, 4.1]	3.4	[0.8, 13.4]
Two racism experiences	3.4**	[1.7, 6.7]	5.7***	[2.3, 14.2]	4.2*	[1.2, 14.5]	4.5	[0.7, 27.2]
Three or more racism experiences	3.9**	[1.8, 8.6]	7.8***	[2.7, 22.5]	4.9***	[2.3, 10.6]	6.7***	[2.3, 19.5]

^a^Adjusted for gender, grade, and sexual orientation. Significance: * = <0.05; ** = <0.01; *** = <0.001. Ref, Reference. For the gender variable, the option “Other” includes (non-binary, another identity, and not sure). For the sexual orientation variable, the option “Other: includes (another identity and not sure). Race is a mutually exclusive variable. Sample sizes vary due to missing data ~ < 5%. Bolded values with asterisks indicate statistical significance.

### 3.6 Black/African American

The adjusted multivariable results also revealed significant associations between one, two, and three or more experiences of racism across most of four mental health outcomes among Black/African American adolescents (Table 4). Black/African American adolescents with three or more experiences of racism showed more robust significant associations with depressive symptoms (aOR: 4.1, 95% CI: 2.1, 8.1), considering suicide (aOR: 2.9, 95% CI: 1.7, 4.9), making a suicide plan (aOR: 3.3, 95% CI: 1.6, 6.9), and attempting suicide (aOR: 4.1, 95% CI: 2.1, 8.1) as compared to fewer experiences with racism. Additionally, across the three racial and ethnic groups, Black/African American adolescents reported significantly more robust association with depressive symptoms.

**Table 4:** Adjusted multivariable logistic regression regarding the association between cumulative experiences of racism and mental health outcomes among black/African American adolescents.

Black/African American mental health outcomes								
Experience of racism variables	Depressive symptoms		Considering suicide		Making a suicide plan		Attempting suicide	
	aOR^a^	95% CI	aOR^a^	95% CI	aOR^a^	95% CI	aOR^a^	95% CI
Experience of racism								
No racism experience	Ref.	Ref.	Ref.	Ref.	Ref.	Ref.	Ref.	Ref.
One racism experience	1.8*	[1.2, 3.0]	1.5	[0.6, 3.4]	2.6*	[1.1, 6.1]	2.3	[0.9, 6.2]
Two racism experiences	4.0***	[2.2, 7.2]	2.3*	[1.2, 4.7]	3.1***	[1.8, 5.6]	2.5	[1.0, 6.3]
Three or more racism experiences	4.1***	[2.1, 8.1]	2.9***	[1.7, 4.9]	3.3**	[1.6, 6.9]	4.1***	[2.1, 8.1]

^a^Adjusted for gender, grade, and sexual orientation. Significance: * = <0.05; ** = <0.01; *** = <0.001. Ref, Reference. For the gender variable, the option “Other” includes (non-binary, another identity, and not sure). For the sexual orientation variable, the option “Other: includes (another identity and not sure). Race is a mutually exclusive variable. Sample sizes vary due to missing data ~ < 5%. Bolded values with asterisks indicate statistical significance.

### 3.7 Hispanic/Latinx

The adjusted multivariable results showed significant associations between one, two, and three or more experiences of racism across all the four mental health outcomes among Hispanic/Latinx adolescents (Table 5). Hispanic/Latinx adolescents with three or more experiences of racism showed stronger associations with depressive symptoms (aOR: 3.8; 95% CI: 2.9, 5.1), considering suicide (aOR: 4.3; 95% CI: 2.8, 6.8), making a suicide plan (aOR: 4.9, 95% CI: 3.3, 7.4), and attempting suicide (aOR: 3.6; 95% CI: 2.2, 5.8) as compared with no experiences with racism. Additionally, across the three racial and ethnic groups, Hispanic/Latinx adolescents reported significantly more robust association with making a suicide plan.

**Table 5:** Adjusted multivariable logistic regression regarding the association between cumulative experiences of racism and mental health outcomes among Hispanic/Latinx adolescents.

Hispanic/Latinx mental health outcomes								
Experience of racism variables	Depressive symptoms		Considering suicide		Making a suicide plan		Attempting suicide	
	aOR^a^	95% CI	aOR^a^	95% CI	aOR^a^	95% CI	aOR^a^	95% CI
Experience of racism								
No racism experience	Ref.	Ref.	Ref.	Ref.	Ref.	Ref.	Ref.	Ref.
One racism experience	1.8***	[1.5, 2.3]	1.7**	[1.2, 2.5]	1.9***	[1.5, 2.4]	1.8**	[1.2, 2.7]
Two racism experiences	3.4***	[2.4, 4.9]	3.8***	[3.0, 4.8]	3.6***	[2.7, 4.8]	3.0***	[2.2, 4.0]
Three or more racism experiences	3.8***	[2.9, 5.1]	4.3***	[2.8, 6.8]	4.9***	[3.3, 7.4]	3.6***	[2.2, 5.8]

^a^Adjusted for gender, grade, and sexual orientation. Significance: * = <0.05; ** = <0.01; *** = <0.001. Ref, Reference. For the gender variable, the option “Other” includes (non-binary, another identity, and not sure). For the sexual orientation variable, the option “Other: includes (another identity and not sure). Race is a mutually exclusive variable. Sample sizes vary due to missing data ~ < 5%. Bolded values with asterisks indicate statistical significance.

## 4 Discussion

This study used state-representative data of Colorado high school-aged adolescents to assess the self-reported prevalence of four experiences of racism, four mental health outcomes, and how cumulative experiences of racism were associated with these mental health outcomes among Asian American, Black/African American, and Hispanic/Latinx adolescents. Our findings demonstrate significant associations between cumulative experiences of racism across most of the mental health outcomes among these racial and ethnicity minority adolescents.

We found that Native Hawaiian and Pacific Islander adolescents reported the highest levels of experiencing racism, though these estimates were accompanied by wide confidence intervals. Correspondingly, Black/African American adolescents reported the next highest levels, with Asian American adolescents reporting somewhat lower experiences of racism. These findings for Black and Asian adolescents in Colorado are consistent with broader research that highlights similar patterns of experiences with racism among these groups (6, 7).

Consistent with previous literature, our study disclosed significant correlations between experiences of racism with depressive symptoms and suicidality (6–9, 11–13, 15). However, our study delved deeper into unpacking the impact of the cumulative effects of multiple experiences with racism on adolescents’ mental health outcomes. We discovered that with each additional experience of racism, Asian American, Black/African American, and Hispanic adolescents were significantly at higher risks of depressive symptoms and suicidality. A comparison across the three racial and ethnic groups in our study reveals distinct patterns in the relationship between experiences of racism and mental health outcomes. Asian American adolescents showed strong cumulative effects of racism, with higher odds in several mental health categories, particularly in considering and attempting suicide, highlighting a pronounced association between racism and mental health risks. For Black/African American adolescents, risk becomes more consistent after two experiences of racism, whereas Hispanic/Latinx adolescents show significant associations even after a single experience, with a strong cumulative effect across outcomes. This suggests that racism may have more immediate impacts for some groups, while repeated exposure may be needed for effects to become consistent in others, potentially reflecting differences in social context, coping resources, or cultural connectedness. These findings highlight the importance of addressing both singular and cumulative experiences of racism in prevention and intervention efforts, with culturally responsive approaches that account for differences in how impacts manifest across groups. These patterns underscore the significant impact of racism on poor mental health outcomes. Generalizability is limited by the Colorado-based sample. Colorado has a smaller Black/African American and Asian American population and a relatively larger Hispanic/Latinx population compared to the U. S., which may shape experiences of racism and access to protective community factors. As a result, these findings may not fully translate to settings with different demographic compositions.

Our results are especially concerning given the well-established association between racism and adverse mental health outcomes. In addition, these results have implications for schools and communities in Colorado. Because repeated experiences of racism were associated with adverse mental health outcomes among high-school adolescents, school-based prevention and intervention efforts may benefit from addressing not only incidents of racism if they occur but also the cumulative impact of everyday experiences of racism. Schools may consider screening for ongoing experiences of racism, strengthening culturally responsive mental health services, implementing anti-racism training for staff and students, and promoting inclusive school climates that reduce discriminatory experiences and behaviors among adolescents. Policies and mental health programs that understand the negative impact of cumulative nature of racism may help schools better identify and support students experiencing racism repeatedly. Greater awareness among clinicians, school-based mental health professionals, and community organizations of the relationship between the impact of cumulative experiences of racism and adolescent mental health may help improve screening, prevention, and culturally responsive support for adolescents experiencing racism-related distress. The rapidly increasing rates of suicide among racial and ethnic minority adolescents makes this connection even more critical to address.

### 4.1 Limitations

This study is not without limitations. First, we were unable to assess the impact of cumulative experiences of racism on mental health outcomes across all the racial and ethnic minority groups in our study due to limited sample sizes across certain groups. Thus, future studies should explore these associations, especially among American Indian/Alaskan Native and Native Hawaiian/Other Pacific Islanders given their high prevalences of experiencing racism and adverse mental health outcomes. As our study population encompasses only Colorado adolescents, our results may yield limited external validity. Additionally, our results may be impacted by social desirability bias as the survey relies on self-reported data. As we used a cross-sectional study design, we cannot infer causality regarding the relationship between cumulative experiences of racism and mental health outcomes. Furthermore, our study did not account for various other experiences of racism or their cultural context, nor did it assess the recurrence, severity, or frequency of these experiences, which may affect the interpretation of the cumulative score. The Multiracial category includes diverse racial identity combinations, and the inability to examine specific multiracial subgroups represents a limitation that may obscure important cultural nuances. Cultural differences in how adolescents interpret and report experiences of racism and emotional distress may influence self-reported measures of racism and mental health outcomes. Additionally, the racism items focused on specific interpersonal experiences of unfair treatment and did not capture broader or culturally specific aspects of racism, which represents a limitation when interpreting how racism may be experienced across different racial and ethnic groups. Finally, associations with mental health and mental health-related outcomes may differ even at lower levels of reported racism, and these patterns may vary across racial groups, suggesting the need for further research.

## 5 Conclusion

This study examined the prevalence of experiences of racism and mental health outcomes and investigated the impact of cumulative experiences of racism on mental health outcomes among Asian American, Black/African American, and Hispanic/Latinx adolescents in Colorado. Our findings demonstrated significant associations regarding this relationship among these three racial and ethnic minority groups and found that all three groups with three or more experiences of racism had significantly higher odds of reporting adverse mental health outcomes.

Findings from this study supports ongoing efforts in schools and community settings for anti-racism policies educational programs and mental health interventions. Recognizing racism as a determinant of poor mental health can help start conversations and mitigate the mental health consequences of experiencing racism. Furthermore, our study highlights the complex nature of racism and underscores the need for future research to investigate its effects on various health outcomes among adolescents.

## Data Availability

The data analyzed in this study is subject to the following licenses/restrictions: Due to privacy-related restrictions, the dataset is not included in the manuscript or Supplementary material. Requests to access these datasets should be directed to emily.fine@state.co.us.
