# Supplementary material for: Cumulative experiences of racism and mental health outcomes in Colorado minority adolescents
Source: Front Public Health. 2026 May 28;14:1781495. doi: 10.3389/fpubh.2026.1781495 (PMC13253283; doi:10.3389/fpubh.2026.1781495)
Supplement: Supplementary file 1 [file Table_1.docx]

**Supplement Table 1: Unweighted Demographic Characteristics of Sample**

| **Demographic Characteristics** | **N** | **%** |
| --- | --- | --- |
| **Race** |  |  |
| American Indian/Alaska Native | 423 | 0.9 |
| Black/African American | 1,621 | 3.2 |
| Asian American | 1,679 | 3.7 |
| Hispanic/Latinx | 9,528 | 19.1 |
| Middle Easter/North African/Arab | 233 | 0.5 |
| Native Hawaiian/Other Pacific Islander | 131 | 0.3 |
| White | 25,150 | 50.3 |
| Another Identity | 309 | 0.6 |
| Multiracial | 9,086 | 18.2 |
| **Gender** |  |  |
| Male | 24,290 | 48.6 |
| Female | 22,693 | 45.4 |
| Other | 2,068 | 4.1 |
| **Grade** |  |  |
| 9^th^-10^th^ grade | 28,292 | 56.6 |
| 11^th^-12^th^ grade | 21,247 | 42.5 |
| **Sexual Orientation** |  |  |
| Heterosexual | 37,932 | 75.9 |
| Sexual Minority | 7,353 | 14.7 |
| Other | 2,420 | 4.8 |
| **Note:** N = Number of individuals. For the gender variable, the option “Other” includes (non-binary, another identity, and not sure). For the sexual orientation variable, the option “Other: includes (another identity and not sure). Race is a mutually exclusive variable. Sample sizes vary due to missing data ~ <5%. | | |
